# Purple of Cassius

**Published:** 1860-11

**Authors:** William Calvert


### PURPLE OF CASSIUS.

BY PROF. WILLIAM CALVERT, D. D. S.

VARIOUS have been the formulas, emanating from different well authenticated and scientific sources, for the preparation of this well known, beautiful, and indispensable pigment..

Much difficulty and uncertainty of success not unfrequently attend the usual methods of procuring it ; and hitherto, it has been considered a matter of such extreme delicacy, that even the most experienced would often fail in obtaining the most desirable results.

Fully aware, as I am, from experience, of the difficulties thus likely to be encountered, I indulge the hope, that in the following, I may place the matter upon such reliable basis, as will lead all whom it may concern to a perfectly uniform and successful termination. No method, I feel safe in assuming, will be found, in all points, equally practicable, at the ame time so completely divested of complexity andresolved to its most simple formula, as the one under consideration.

The production of this vitrifiable pigment calls into requisition the *pure* metals, *gold* and *tin ;* and upon their proper relation and combination, together with corresponding accuracy in manipulation, depend the equable results.

For its preparation, then, dissolve forty-eight (48) grains of pure gold in about three fluid drams (F ʒiij) of aqua-regia, composed of hydrochloric acid, 21° B., *two parts*, and nitric acid, 38° B., *one part.* When the gold is all dissolved, pour the solution into a quart precipitating glass, or other suitable vessel, and add to it from eight to twelve ounces of water. Then dissolve thirty-five (35) grains of pure tin in a similar quantity of hydrochloric acid, with a very slight addition of nitric acid. Notwithstanding hydrochloric acid is invariably given by authors as the proper solvent for this metal, (tin), still it is a *fact*, that needs only trial to make it apparent that a minimum quantity of nitric acid, in combination with the hydrochloric, will greatly hasten a solution of the metal. When the tin is dissolved, pour into a vessel, as in case of the gold, and add a like quantity of water to it.

Next prepare a strong solution of carbonate of potassa, in water ; and of this, add to each of the dilute metallic solutions, in small quantities, and as near simultaneously as possible—shaking so as to thoroughly commingle them—until one of two things is observable, viz : either a neutral solution, which may be determined by suitable test papers, (litmus and turmeric, ) or till there is a slightly visible change in the color of the solutions, indicating the near approach of precipitation ; the one containing the gold showing a greenish tinge, and the other, (tin,) a whitish, or milky hue. Should the alkaline solution be too largely added to either, evidently resulting in precipitation, then a small quantity of the acid (solvent of the particular metal,) should be added. If more acid is supplied than is necessary to produce the desired condition, the alkaline solution is again to be very sparingly made use of. Having now a large clean glass, or porcelain vessel at hand, at the moment that either of these conditions becomes apparent, the two solutions, at the same moment and unitedly, should be poured into it, when, upon the instant of union, a combination ensues, yielding the desired purple.

This precipitate must now be washed thoroughly, and then dried. The washing may be most conveniently conducted in the following manner: after the precipitate has settled, the supernatant liquor should be decanted, as closely as possible, so as not to lose any of the pigment, and then fresh *clean* luke-warm water employed, filling the vessel, and permitting it to remain until the settling is again accomplished, when the water should be either poured off, or removed by means of a glass syphon. The addition of water, and the decanting of the same, should be repeated several times. After the last decantation the precipitate, with the residue of water, should be turned into an evaporating dish, which should be placed upon a sand bath, or some other convenient and suitable place, and the water slowly evaporated, thus leaving a brownish purple residue—*the Purple of Cassius*, in a state of preparation, either for the manufacture of gum enamel, or other appropriate uses.

